# Survival and Failure Outcomes in Primary Thyroid Lymphomas: A Single Centre Experience of Combined Modality Approach

**DOI:** 10.1155/2013/269034

**Published:** 2013-09-12

**Authors:** Ritesh Kumar, Divya Khosla, Narendra Kumar, Sushmita Ghoshal, Anjan Bera, Ashim Das, Suresh Chander Sharma

**Affiliations:** ^1^Department of Radiotherapy and Oncology, Regional Cancer Centre, Postgraduate Institute of Medical Education and Research, Chandigarh 160012, India; ^2^Department of Pathology, Postgraduate Institute of Medical Education and Research, Chandigarh 160012, India

## Abstract

Primary thyroid lymphoma (PTL) is a rare malignancy and represents 2%–5% of all thyroid malignancies and 1%–2.5% of all malignant lymphomas. We present our institute's experience in combined modality management of 16 successive patients of PTL treated from 2005 to 2010. The median age of the patients was 56.0 years. Five patients were males, and 11 patients were females. An enlarging thyroid mass was the most common presenting symptom. 14 patients had diffuse large B-cell lymphoma, and 2 patients had follicular lymphoma. The most common stage of presentation was stage II comprising 6 (37.5%) patients. All patients received CCT, and only 12 patients received involved field RT with a median dose of 36.0 Gy. 10 patients (62.5%) had CR, and 6 patients (27.5%) had PR. Eight patients had disease progression in subsequent followup and this included the initial 6 patients with PR. The 5-year DFS was 40.0%, and median DFS was 47 months. The 5-year OS was 41.0%, and median OS was 51 months. Most common presentation in our series was locally advanced tumors. Most of these patients require combined modality management. Risk-adapted and multimodality approach is the need of the hour to achieve good control rates while minimizing treatment related toxicity.

## 1. Introduction 

Primary thyroid lymphoma (PTL) is a rare malignancy and represents 2%–5% of all thyroid malignancies 1%–2.5% of all malignant lymphomas [1, 2]. This rare disease usually affects middle- to older-aged individuals with a female predilection and presents with a rapidly enlarging anterior neck mass with or without cervical lymphadenopathy, often leading to compressive symptoms [3, 4]. PTL is associated with Hashimoto's thyroiditis, and the relative risk of developing malignant lymphoma of thyroid, is greater in patients with Hashimoto's thyroiditis [5, 6]. Most thyroid lymphomas are B-cell type non-Hodgkin's lymphomas (NHL), and Hodgkin's and T-cell lymphomas are extremely rare [7–10]. The optimal management strategy for PTL remains somewhat controversial. Because of the rarity of PTL, no randomized control trial has compared the efficacy of multimodality versus single modality treatment. Radiotherapy (RT) is a mainstay of treatment as PTL is highly sensitive to radiation. Systemic chemotherapy (CCT) has diminished the likelihood of local and systemic relapses; combination CCT is generally considered, followed by involved field radiotherapy (IFRT). We herein report our institutional experience of 16 successive patients of PTL being treated from 2005 to 2010.

## 2. Material and Methods

### 2.1. Patient Population and Initial Evaluation

We retrospectively reviewed the patients of primary thyroid lymphomas (PTL) from January 2005 to December 2010 treated in our institute. Total number of patients was 16. We reviewed the records of these patients to extract the following information: age, sex, clinical symptoms, histology, radiology (CT/MRI), tumor extent, chemotherapy regimens and doses, radiation (technique, total dose, dose per fraction, and number of fractions), toxicity, response, recurrence, progression, metastases, and death. Staging investigations included medical history and physical examination, complete hematological profile, blood chemistry, chest radiograph, contrast enhanced computed tomography (CECT) of neck, chest, abdomen, and pelvis, and bone marrow aspiration and biopsy.

### 2.2. Treatment

Surgery was limited to biopsy for histopathological diagnosis. After initial FNAC diagnosed thyroid lymphoma, all patients underwent core biopsy for exact histopathological characterization and flow cytometric studies. CCT and RT were used in the treatment. CCT was given with either Cyclophosphamide, Doxorubicin, Vincristine, and Prednisolone (CHOP) or (CVP) Cyclophosphamide, Vincristine, and Prednisolone regimen in standard doses at three weekly intervals. RT was delivered in conventional 1.8–2 Gy per fraction. RT planning evolved with time and expertise, and patients were planned with two-dimensional (2D), three-dimensional conformal (3DCRT), and intensity modulated (IMRT) techniques. 

### 2.3. Followup

The period between the first complaint and diagnosis was registered as symptom duration. Survival, recurrence, and progression information were collected through chart review, patient, or relative contact. Response evaluation was noted both clinically and radiologically, and RECIST criteria were applied [11].

### 2.4. Statistical Analysis

SPSS v 15 was used for statistical analysis. The Kaplan-meier survival analysis was done for analyzing disease-free-survival (DFS) and overall survival (OS) [12]. 

## 3. Results

### 3.1. Patient Characteristics

Patient characteristics are summarized in Table 1. Between January 2005 and December 2010, 16 patients of PTL were registered in our department. The median age of the patients was 56.0 years and ranges from 24 years to 68 years. five patients (31.3%) were males and 11 patients (68.7%) were females. The median duration of symptoms was 6 months. An enlarging thyroid mass was the most common presenting symptom manifesting in all of the patients followed by aerodigestive tract symptoms, including dyspnea and dysphagia. B-symptoms were present in 7 (43.75%) patients and neck lymphadenopathy was present in 12 (75.0%) patients. None of the patients had thyroiditis. On histopathological analysis, 14 patients (87.5%) had diffuse large B-cell lymphoma (DLBCL) and 2 patients (12.5%) had follicular lymphoma (FL). The most common stage of presentation was stage II comprising 6 (37.5%) patients followed by stages I, III, and IV comprising 4 (25.0%), 3 (18.75%), and 3 (18.75%) patients, respectively. Out of the three stage IV patients, two had bone marrow involvement, while one had liver involvement. 

### 3.2. Treatment Details (Table 2)

Treatment modalities consisted of CCT and RT. All patients received CCT, 15 (93.75%) patients with CHOP regimen and one patient with CVP regimen. Median number of cycles was six. 12 patients received involved field localized RT with a median dose of 36.0 Gy (range 30–40 Gy) in 1.8–2 Gy per fraction. RT was delivered using 2D RT in 7 patients (43.75%), and 3DCRT in 5 (31.25%) while 2 patients (12.5%) did not receive RT. The 2 patients who did not receive RT were young patients with stage II FL histology. 

### 3.3. Treatment Toxicity and Compliance

All patients completed treatment with no significant toxicity or treatment interruption. RT toxicity occurred in 12 patients (75.0%) in the form of grade 1-2 dermatitis and mucositis, and there was no grade 3 or higher toxicity. CCT toxicity was seen in 7 patients in the form of grade 1-2 hematological toxicity. 

### 3.4. Clinical Outcomes (Table 3)

**Figure 1:**
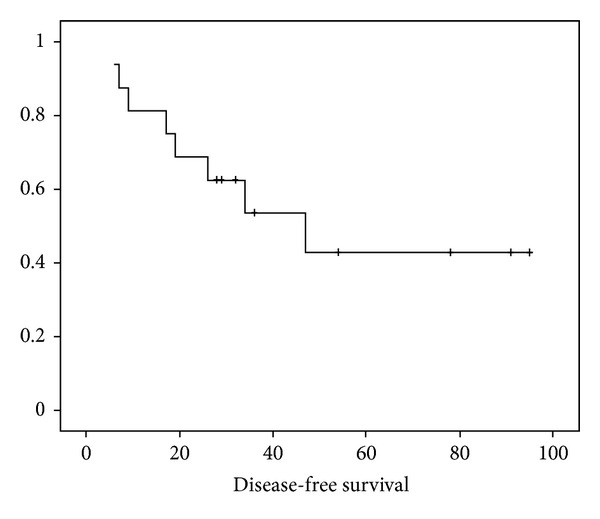
The Kaplan-Meier curve showing disease free-survival (DFS).

**Figure 2:**
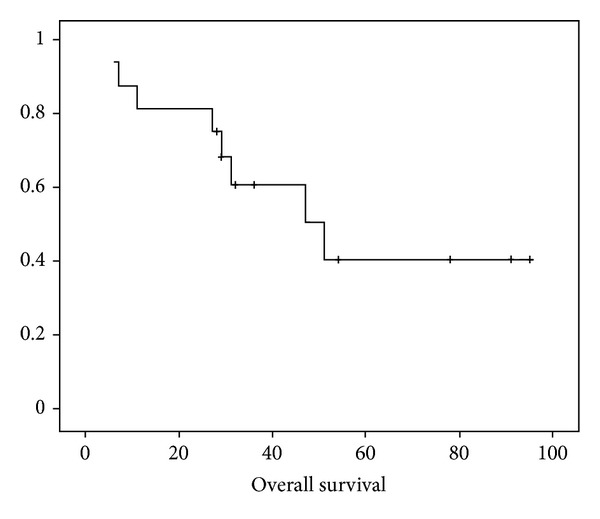
The Kaplan-Meier curve showing overall-survival (OS).

After treatment completion, patients were assessed for response both clinically and radiologically. 10 patients (62.5%) were asymptomatic and 6 patients (27.5%) had significant improvement in symptoms. As per the RECIST criteria, 10 patients (62.5%) had CR and 6 patients (27.5%) had PR. Eight patients had disease progression in subsequent followup, and this included the initial 6 patients with PR. The other 2 patients who initially had CR had recurrence in retroperitoneal lymph node (RPLN) and brain respectively. Out of the eight patients who had disease progression, 5 received second line CCT with MIME regimen comprising mitoxantrone, ifosfamide, and etoposide in standard doses. One patient with recurrence in brain received whole brain RT in doses of 30 Gy in 10 fractions. Median duration of followup was 40 months (range 10–95). The 5-year disease free-survival (DFS) of all patients was 40.0% (Figure 1) and median DFS was 47 months. The 5-year overall survival (OS) was 41.0% and median OS was 51 months (Figure 2). On subgroup analysis, the stagewise median survival was 65 months, 54 months, 29 months, and 8 months for stages I, II, III, and IV, respectively.

## 4. Discussion

Primary thyroid lymphoma (PTL) is a rare malignancy and represents 2%–5% of all thyroid malignancies, 1%–2.5% of all malignant lymphomas, and 2.5%–7% of all extranodal lymphomas [1, 2]. PTL is commonly seen in middle to older aged women and usually presents as a painless mass in the thyroid gland or diffuse enlargement that causes symptoms related to compression, such as hoarseness, dysphagia, and dyspnea [3, 4]. In our series, PTL was more common in females (68.7%), and the most common presentation was diffuse enlargement of thyroid gland followed by compressive symptoms. The presence of B-symptoms is uncommon. PTL is associated with Hashimoto's thyroiditis, and the relative risk of developing malignant lymphoma of thyroid has been estimated to be 40–80 times greater in patients with Hashimoto's thyroiditis [5, 6]. However, none of the patients in our series had associated thyroiditis. 

Most thyroid lymphomas are B-cell type non-Hodgkin's lymphomas (NHL), and Hodgkin's and T-cell lymphomas are extremely rare [7–10]. The most common B-cell NHL is DLBCL and thyroid DLBCL at a localized stage generally has a good prognosis, but it is heterogenous and sometimes has a poor prognosis [13]. Fourteen patients (87.5%) had DLBCL and 2 patients (12.5%) had FL in our series. 

Ann Arbor staging system is used for staging as in systemic NHL [14]. Most of the patients present with stage I or II disease [15]. In our series, the most common stage of presentation was stage II comprising 6 (37.5%) patients followed by stages I, III, and IV comprising 4 (25.0%), 3 (18.75%) and 3 (18.75%) patients. Out of the three stage IV, patients, respectively, two had bone marrow involvement, while one had liver involvement. 

The optimal management strategy for PTL remains somewhat controversial. Because of the rarity of PTL, no randomized control trial has compared the efficacy of multimodality versus single modality treatment. Earlier, local surgery was the primary management strategy because of the difficulty in distinguishing histologically between malignant lymphoma and anaplastic thyroid carcinoma, especially based on pre-operative biopsy [16, 17]. Now, diagnosis can be accomplished using fine needle aspiration biopsy (FNAB) or by core or open biopsy [18, 19].

RT is mainstay of treatment as PTL is highly sensitive to radiation. The RT doses delivered varied from 30 to 50 Gy as reported in different series [20, 21]. To diminish the likelihood of local and systemic relapses, combination chemotherapy is generally considered, followed by involved field radiotherapy (IFRT) [22, 23]. The CCT regimens used commonly are CVP, CHOP, and R-CHOP as in systemic NHL [24, 25]. In the literature, the 5-year OS rates range from 35% to 100%. Ha et al. showed 5-year OS and DFS of 64% and 76% in a retrospective series and concluded that localized PTL has good prognosis when managed with combined modality treatment [26]. Matsuzuka et al. reported a 100% OS rate at 8 years for a subset of patients treated with CCT and RT [27]. In a review of 211 patients with PTL, Doria et al. demonstrated that addition of CCT to RT significantly lowered the distant metastases and overall recurrence rates [22]. The 5-year OS in our series was 41%, and these slightly inferior results may be attributed to the 6 patients (37.5%) who had stage III, and IV disease

The most important prognostic factors influencing the outcome are age, stage, histopathological subtype, grade, RT, and CCT [28–32]. Local recurrences and distant metastases may develop long after the initial treatment, sometimes after several years, underlining the need for long term followup. 

The management of PTL is a paradigm of cooperation between clinicians, surgeons, and pathologists from establishing diagnosis to organizing the therapeutic strategy. With newer targeted chemotherapeutic agents, radiation techniques, diagnostic and imaging facilities, there is a significant improvement of therapeutic standard, and PTL represents a model of therapeutic implementation and achievement in oncology. 

## Figures and Tables

**Table 1:** Patient characteristics.

Total number of patients	16
Age (in years)	
Median	56.0
Range	24–68
Sex	
Male	5 (31.3%)
Female	11 (68.7%)
Stage	
I	4 (25.0%)
II	6 (37.5%)
III	3 (18.75%)
IV	3 (18.75%)
B-symptoms	
Present	7 (43.75%)
Absent	9 (56.25%)
Neck node	
Present	12 (75.0%)
Absent	4 (25.0%)
Thyroiditis	
Present	0 (0.0%)
Absent	16 (100.0%)
Histology	
DLBCL	14 (87.5%)
FL	2 (12.5%)

**Table 2:** Treatment details.

Treatment approach	
CCT + RT	14 (87.5%)
CCT alone	2 (12.5%)
CCT regimen	
CHOP	15 (93.75%)
CVP	1 (6.25%)
RT	
Dose (median)	36.0 Gy
Dose (range)	30–40 Gy

**Table 3:** Response evaluation at treatment completion.

Clinical response	
Asymptomatic	10 (62.5%)
Improved	6 (27.5%)
Radiological response	
CR	10 (62.5%)
PR	6 (27.5%)
